# Spheroid-Formation (Colonosphere) Assay for in Vitro Assessment and Expansion of Stem Cells in Colon Cancer

**DOI:** 10.1007/s12015-016-9664-6

**Published:** 2016-05-20

**Authors:** Sameerah Shaheen, Mehreen Ahmed, Federica Lorenzi, Abdolrahman S. Nateri

**Affiliations:** Cancer Genetics and Stem Cell Group, Cancer Biology Unit, Division of Cancer and Stem Cells, School of Medicine, University of Nottingham, Nottingham, NG7 2UH UK

**Keywords:** Colonosphere, In vitro assay, Colorectal cancer, Cancer stem cell, Immunofluorescence, Self-renewal, Differentiation

## Abstract

Colorectal cancers (CRCs) form a disorganized hierarchy of heterogeneous cell populations on which current chemotherapy regimens fail to exert their distinctive cytotoxicity. A small sub-population of poorly differentiated cancer stem-like cells (CSCs), also known as cancer initiating cells, may exhibit embryonic and/or adult stem-cell gene expression signatures. Self-renewal and survival signals are also dominant over differentiation in CSCs. However, inducers of differentiation exclusive to CSC may affect cellular pathways required for the formation and progression of a tumor, which are not utilized in normal adult stem-cells. Nevertheless, assays for targeting CSCs have been hindered by expanding and maintaining rare CSCs in vitro. However, CRC-CSCs are able to form floating spheroids (known as colonospheres) 3-dimentinionally (3D) in a serum-free defined medium. Therefore, great efforts have been paid to improve colonosphere forming assay as a preclinical model to study tumor biology and to conduct drug screening in cancer research. The 3D-colonosphere culture model may also represent in vivo conditions for the spontaneous aggregation of cancer cells in spheroids. This protocol describes the development of an enrichment/culture assay using CRC-CSCs to facilitate colorectal cancer research through immunofluorescence staining of colonospheres. We have developed colonospheres from HCT116 CRC cell line to compare and link CRC-CSC markers to the NANOG expression level using an immunofluorescence assay. Our data also show that the immunostaining assay of colonosphere is a useful method to explore the role and dynamics of CRC-CSCs division between self-renewal and cell lineage differentiation of cancer cells. In principle, this method is applicable to a variety of primary cells and cell lines of epithelial origin. Furthermore, this protocol may also allow screening of libraries of compounds to identify bona fide CRC-CSC differentiation inducers.

## Introduction

CSCs have garnered substantial interest over the past few years. However, specific cell markers enabling the identification of CSCs in most tumor entities, and to provide a reliable in vitro model suitable for CSC-studies are still lacking. Therefore, to explore the self-renewal and differentiation properties of CSCs in vitro, experimental assays must reliably be able to distinguish CSCs and their progeny.

Human CRCs are composed of a heterogeneous mixture of cancer cells [1, 2]. Minor proportions of these CRC cells strongly resembled a small sub-population of self-renewing and poorly differentiated CSCs (also known as cancer initiating cells) [3, 4]. CSCs evade conventional drugs, and significantly contribute to adverse survival rates [5, 6]. Recently, it was also reported that CSCs sub-population exhibit an embryonic stem cell gene expression signature [3, 7]. We and others have reported the expression of embryonic proteins, including carcino-embryonic-antigen (CEA), alkaline-phosphatase, and NANOG, in CRC and other cancers, which may contain undifferentiated multipotent cancer cells [3, 7–10].

Current studies delineated a physiological balance of self-renewal versus differentiation potential in normal and CSC cells [11, 12]. Self-renewal and survival signals are dominant over differentiation counterparts in CSCs [4, 13]. These suggest that an overexpression of embryonic stem cell associated proteins such as NANOG may be an essential modulator of cancer cell drug-resistance mechanisms, which also contributes to prevent differentiation in CSCs.

It was recently reported that CSCs can be grown to form floating spheroids in vitro when plated in limited numbers in a serum-free medium supplemented with growth factors [14]. Spheroids are characterized by their well-rounded shape, presence of cancer cells, and their capacity to promote in vitro expansion of CSCs compared to the bulk of tumors [15, 16]. This helps to culture and study CSCs in spheroid-forming assays as reported for neurospheres [17, 18], mammospheres [19, 20] and colonospheres [21, 22]. Moreover, the spheroid-forming assay has gained wide popularity in CSC research as it allows evaluating self-renewal and differentiation abilities at the single-cell level. Using this approach, we expanded colon CSCs by generating colonospheres from CRC cell line (HCT116) (Notes 4.1 and 4.2), which stably expressed exogenous NANOG (HCT116-GFP/NANOG) [3, 10] (a CSC signature), as well as from parental CRC (HCT116-GFP) cells and used them to isolate potential colorectal CSCs.

In the present study, we cultured colonospheres on coverslip and performed immunofluorescence assay for the common markers of stemness and differentiation, CD44 [23] and MUC2 [24, 25], respectively. CD44 is a hyaluronic acid receptor and a transmembrane glycoprotein that regulates several processes important for tumor progression, including proliferation, adhesion and differentiation [23]. Interestingly, CD44 is not only a marker for colorectal CSCs, but is also a critical molecule for modulating stemness in CSCs [24, 26] and plays a functional role in cancer initiation [27]. MUC2 is a large glycoprotein and main component of the protective mucous layer in the intestine [28]. Moreover, MUC2 is a goblet cell marker associated with epithelial differentiation [25, 29, 30].

Furthermore, this work showed that gain of a pluripotency marker and loss of a differentiation marker may alter not only the exclusive CRC-CSCs signaling/pathway(s) but also those unique cell-surface markers that are required for targeting CRC-CSCs in order to improve colorectal cancer therapy.

## Materials

The human CRC cell line HCT116 (Notes 4.1, 4.2 and 4.3), was originally purchased from the American Type Culture Collection (ATCC) (Cat#ATCC-CCL-247).

DMEM/F-12 (Dulbecco’s Modified Eagle Medium/Nutrient Mixture F-12) is a basal medium used for the growth of HCT116 cells and colonospheres, were purchased from Life Technologies (Cat#11,320–074).

Penicillin (100/ units/ml) and Streptomycin (100 mg/ml) antibiotics are used to prevent bacterial contamination in cell cultures, were purchased from Invitrogen Corporation (Cat#15,140–122).

Bioactive recombinant human FGF basic 146 aa (rhFGF) was purchased from R&D systems and stored at −20 °C after use (Cat#233-FB).

Recombinant mouse epidermal growth factor (EGF) was purchased from Invitrogen and stored at 4 °C after use (Cat#PMG8043).

N-2 Supplement (×100) was purchased from Life Technologies and recommended for growth of colonsphere (Cat#17,502–048).

Trypsin-EDTA (0.05 %), phenol red was purchased from Thermo Fisher Scientific (Cat#25,300–054).

Coverslips (size: 22 × 26 mm) was purchased from VWR Collection(Cat#631–0131).

ProLong Gold antifade reagent with DAPI was purchased from Life Technologies (Cat#P36962).

Goat anti-rat secondary antibody, DyLight was purchased from Bethyl Laboratories (Cat#A110-105D4).

Monoclonal rat anti-CD44 antibody was purchased from Millipore(Cat#MAB2137).

Rabbit polyclonal anti-CD44 antibody was purchased from Millipore (Cat#MAB2137).

Phosphate buffered saline (PBS) (Cat#D8537), Poly-L-lysine (Cat#P4707), RPMI-1640 (Roswell Park Memorial Institute) (Cat#R0883), FBS (Fetal Bovine Serum) (Cat#F7524) all purchased from Sigma Aldrich.

## Methods

### Preparation of Poly-L-Lysine-Coated Coverslips in Sterile 6-well Cell Culture Plate

Immerse your sterile uncoated coverslips of 22 × 26 mm thickness in 70 % ethanol for 30 min. Wash coverslips twice in 1 ml sterile phosphate buffered saline (without calcium/magnesium chloride) (PBS) for 5 min each.

Place the coverslips in the wells of 6-well cell culture plate using sterile forceps and wash coverslips one time in PBS for 5 min.

Coat coverslips with 1.5 ml of the commercially premade 0.01 % poly-L-lysine solution and incubate the plate 1 h at room temperature in hood.

Wash the coated coverslips with 1 ml sterile PBS per well. Air-dry the coverslips and keep the 6-well plate uncovered.

Once dried, label the plate with the name of cell lines and date. The 6-well plate is now ready with poly-L-lysine-coated coverslips for the immediate seeding of cells (Note 4.7).

### Preparing Stem Cell Medium

Use the commercially available Dulbecco’s Modified Eagle Medium/F-12 Nutrient Mixture (Ham) + L-Glutamine (DMEM/F-12) medium. Optionally supplement with 1 U/ml penicillin/streptomycin antibiotics.

Add growth factors such as human recombinant basic fibroblast growth factor (bFGF) at 10 ng/ml concentration, human recombinant epidermal growth factor (EGF) at 10 ng/ml concentration and 100 X N-2 supplements. Add growth factors just before use (fresh) (Note 4.6).

### Generating Colon Cancer Spheroids (Colonospheres)

Grow and maintain HCT116-GFP (control cell line) and HCT116-GFP/NANOG (experimental cell line) in a Roswell Park Memorial Institute (RPMI) medium, supplemented with 10 % heat inactivated fetal bovine serum (FBS) and 2 mM L-glutamine in 75 cm^2^ tissue culture flasks.

Incubate flasks in a humidified incubator at 37 °C and 5 % CO_2_. Change the medium every 3–4 days. This medium does not contain penicillin/streptomycin.

Monitor the cells under an inverted microscope with a 10× magnification. Once the cells are 60–80 % confluent, aspirate media and wash with 3 ml pre-warmed and sterile PBS. Add 2 ml of 0.05 % trypsin ethylenediaminetetraacetic acid (EDTA) solution and incubate in 37 °C incubator for 5 min.

Neutralize trypsin by adding 6 ml of complete culture medium (described in 3.3.1) to each flask and pipette cells up and down to obtain single cell suspension. Transfer the complete cell suspension to a 15 ml labelled Falcon tube.

Centrifuge cells for 5 min at 350 g at room temperature aspirate the supernatant and resuspend the pellet in 6 ml of sterile PBS. Avoid generating bubbles when mixing cells.

Count cells with hemocytometer and adjust accordingly the number of cells to 3000 per 2 ml of complete stem cell medium (described in section 3.2), per well of the 6-well plate.

Seed cells onto the poly-L-lysine-coated coverslips (described in section 3.1) and incubate cells under standard conditions at 37 °C and 5 % CO_2_ for two weeks. Colonospheres with rigid margin will be observed by day 14.

Replace medium with freshly prepared complete stem cell medium (described in section 3.2) every 3–4 days. Change medium in a very gentle manner as the colonospheres grow as floating spheroid colonies (Notes 4.4 and 4.8).

### Immunofluorescent Staining of Colonospheres

Fixing and blocking of colonospheres: aspirate gently the stem cell medium from the well edges of the 6-well plate and empty into a hazardous waste container after 14 days of culture. Wash colonospheres one time in 1 ml PBS.

Prepare 4 % paraformaldehyde (PFA) in PBS: add very gently 800 μl of 4 % PFA to the side of the well and incubate for 30 min at room temperature. Caution: Paraformaldehyde is moderately toxic by skin contact. Gloves and safety glasses should be worn and solutions should be made inside a fume hood.

Wash the fixed cells twice in 1 ml PBS, following aspiration and emptying the collected PFA into a hazardous waste container, and incubate for 5 min at room temperature. Remove the PBS after 5 min.

Add 800 μl of 0.5 % Triton x-100 (diluted in PBS) to permeabilize colonospheres at room temperature to each well. Aspirate the Triton x-100 after 5 min and wash with PBS and place them at room temperature for 5 min.

Block permeabilized colonospheres by adding 800 μl of 1 % bovine serum albumin (BSA) in PBS to each well and leave the plate for 30 min at room temperature. Tilt the 6-well plate to approximately 45° and aspirate BSA/PBS from each well. Colonospheres are now ready for immunofluorescent staining.

Immunofluorescence Staining for CD44 and MUC2 (Notes 4.5 and 4.9).

Dilute the primary anti-CD44 antibody to 1:200 and the anti-MUC2 antibody to 1:100 (Table 1) in 1 % BSA blocking buffer.

**Table 1:** List of key materials used for colonosphere forming assay

Material	Company	Catalogue Number	Comments/Description
DMEM/F-12	Life Technologies	11,320–074	Warm it up at 37 °C water bath before use
Penicillin/Streptomycin	Invitrogen	15,140–122	Penicillin (100 units/ml), Streptomycin (100 mg/ml)
Human Recombinant FGF-basic	R&D system	233-FB	Can be stored at −20 °C after use
Mouse Recombinant EGF	Invitrogen	PMG8043	Can be stored at 4 °C after use
N-2 Supplement	Life Technologies	17,502–048	Can be stored at 4 °C after use
Trypsin	Gibco	25,300–054	
HCT 116	American Type Culture Collection	ATCC-CCL-247	Frozen
Coverslips	VWR Collection	631–0131	Size of coverslips is 22 × 26 mm
PBS	Sigma Aldrich	D8537	
Poly-L-lysine	Sigma Aldrich	P4707	
RPMI-1640	Sigma Aldrich	R0883	
FBS	Sigma Aldrich	F7524	
ProLong Gold antifade reagent with DAPI	Life Technologies	P36962	Store at 2–8 °C or freeze at −5 to −30 °C
CD44 antibody	Millipore	MAB2137	
MUC2 antibody	Santa Cruz	sc-15,334	
Goat anti-rat secondary antibody, DyLight 594	Bethyl Laboratories	A110-105D4	
Donkey anti-rabbit secondary antibody, Alexa Fluor 594 conjugate	Life Technologies	A21207	
BSA	Santa Cruz	sc-2323 A	
PFA	Fisher Scientific	F/1501/PB17	Toxic to skin contact
Tween-20	Sigma Aldrich	P1379	

Remove blocking solution, gently add 400 μl of the diluted primary antibody and incubate for 1 h at 37 °C dry incubator.

Wash each well with 800 μl of washing buffer (1 % BSA, 0.1 % Tween-20 in PBS), twice for 10 min at room temperature.

Dilute the secondary antibody in 1 % BSA blocking buffer; a 1:200 dilution of goat anti-rat secondary antibody for CD44 (Table 1) and a 1:500 dilution of donkey anti-rabbit secondary antibody for MUC2 (Table 1).

Remove washing buffer and add 500 μl of the secondary antibody to each well and incubate for 1 h in dark room to avoid bright light exposure.

Repeat washing steps (3.4.6.3) three times with at least 5 min of incubation time in each wash. Avoid exposing the plate to bright light during washing steps. After the last wash, leave about 200 μl of washing buffer in each well.

Label 6 microscopic slides (1–2 mm thickness) with the name of the cell line and the marker.

Add a small drop (~10 μl) of anti-fade reagent mounting medium with 4′,6-diamidino-2-phenylindole (DAPI) on the middle of microscopic slides and avoid bubble formation.

Use sharp forceps to carefully lift the cover slips from the 6-well plate. Turn the coverslip gently and place the coverslip (with the spheroid side down) on the DAPI mounting medium containing the microscopic slide.

Repeat step 3.4.6.9 with all coverslips. Seal with nail varnish and let the slides dry for 15 min. Place slides in a tray or a slide box and incubate them overnight in a dark place or cover with aluminium foil at 4 °C.

Capture the fluorescence images of stained colonospheres using a fluorescence microscope with 40× or 60× magnifications [31].

## Notes

CRC cell line HCT116 (S45 β-catenin mutant) overexpressing the green fluorescent protein (HCT116-GFP) and/or GFP-fused NANOG protein (HCT116-GFP/NANOG) cell lines are used [3].

Cells were confirmed to be mycoplasma negative. All cell culture is undertaken in sterile mammalian tissue culture hood.

**Fig. 1:**
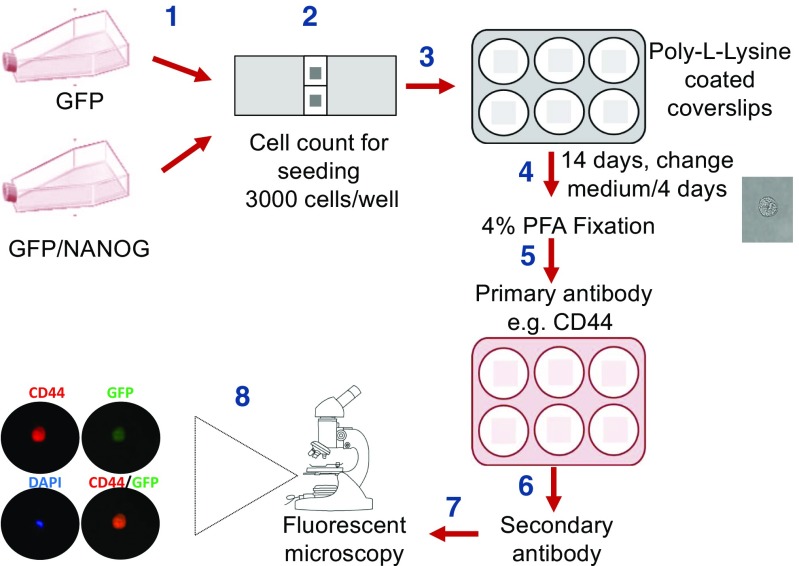
Workflow of the protocol: a schematic illustration of colonosphere formation, fixation and staining for markers. HCT116 cells (GFP vs GFP/NANOG) were seeded on sterile coverslips coated with poly-L-lysine and placed into 6-well culture plate at a density of 3 x 10^3^ cells/well. Colonospheres formed after 14 days were fixed with 4 % PFA for immunofluorescence studies of different markers, such as CD44 and MUC2. The stained colonospheres were subjected to fluorescence microscopy analysis

**Fig. 2:**
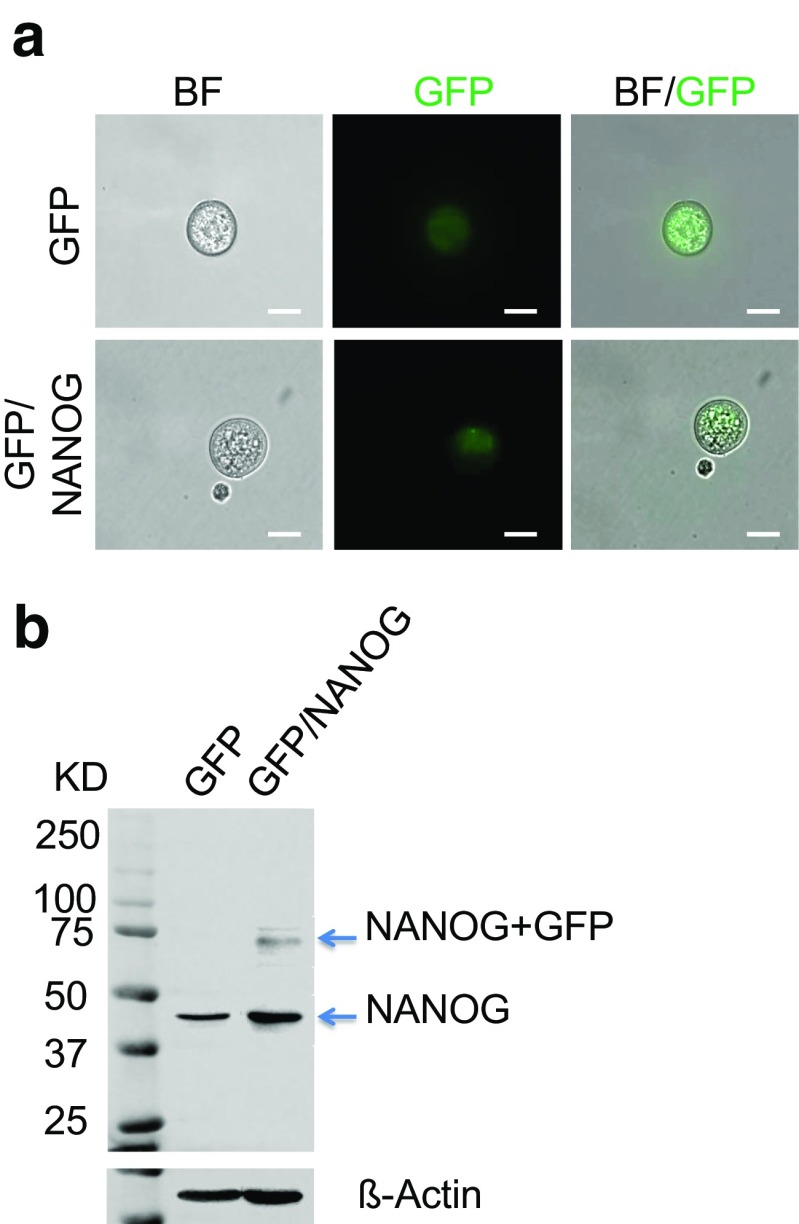
Typical round colonospheres derived from GFP and GFP/NANOG expressing HCT116 cells. Cells grown on top of each other and that formed clusters with a rounded shapes and rigid margins in a three dimension (3D) culture. **a** The left panel shows the bright field (BF) view, the middle panel shows the expression of GFP and the right panel shows merged images of BF and GFP for the colonospheres. *Scale bars* =250 μm. **b** Whole cell lysates isolated from HCT116 cells (GFP and GFP/NANOG) were Western blotted using antibodies against NANOG and the loading control β-actin

HCT116 CRC cell provide an excellent in vitro model to dissect the molecular mechanisms that control the biology of CRC [25, 31]. Establishment of the colonosphere formation assay using HCT116 CRC cell lines (Fig. 1) indicated that a small subpopulation of CRC cells (2–4 %) can be maintained in stem cell medium to form colonospheres (Fig. 2a and data not shown). These colonospheres are derived from a single cancer cell grown in stem cell specific medium [32–35]. However, not every cell has the ability to survive and proliferate in such environment [36]. This displays the efficiency of this assay to accurately estimate the self-renewal potential of CRC-CSCs. Furthermore, for a comparative analysis of the relationship between CRC-CSCs self-renewal and differentiation, colonospheres can be cultured on coverslip and examined for the expression of stemness and differentiation markers as illustrated in Fig. 1, e.g., CD44 and MUC2 respectively.

**Fig. 3:**
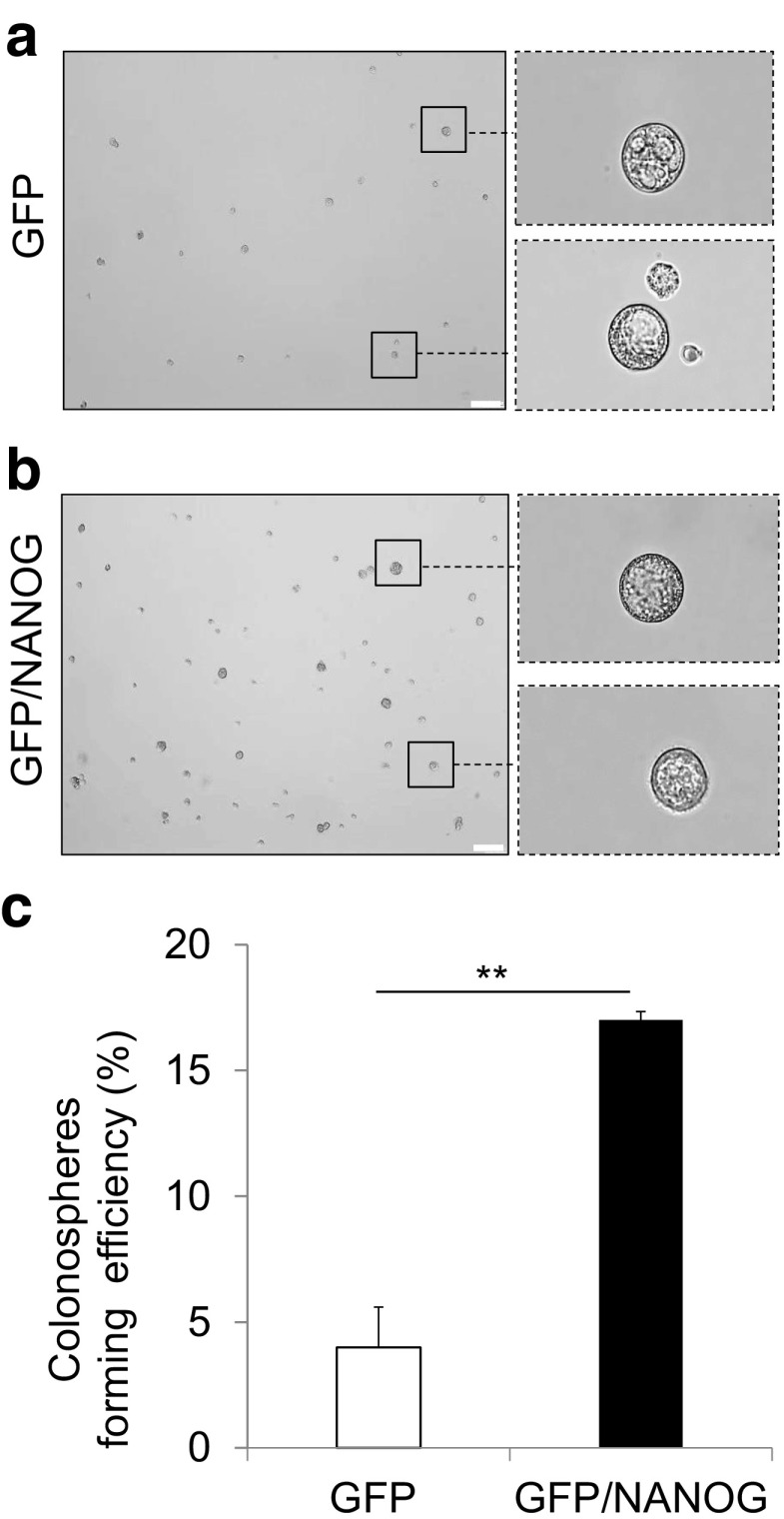
Growth of HCT116 colonospheres (GFP and GFP/NANOG) under 3D culture in spheroid-medium prior to immunofluorescence staining. Colonosphere formation is analyzed after 2 weeks; (**a**) HCT116 GFP cell line and (**b**) HCT116 GFP/NANOG cell line. *Scale bars* =250 μm, 10× magnification. **c** Quantitative data showing the growth of colonosphere formation efficiency in GFP/NANOG versus GFP cells. Data represent mean ± SD *n* = 3 independent experiments. Student *t* test was used to calculate *p* values (*P < 0.05, **P < 0.01, ***P < 0.001)

The colonospheres formed typical circular structure (Fig. 2a) and within a single spheroid, the cells appeared fused together resembling a solid cellular cluster making it hard to distinguish as individual cells [36, 37]. Moreover, the size of spheroids ranges from less than 50 μm to 250 μm (Fig. 3) [38, 39]. Next, the influence of NANOG overexpression on the efficiency of colonosphere formation was evaluated and compared with HCT116-GFP cells and GFP/NANOG cells, which exhibited an increase in spheroid formation by 14–17 %, as shown in Fig. 3c.

**Fig. 4:**
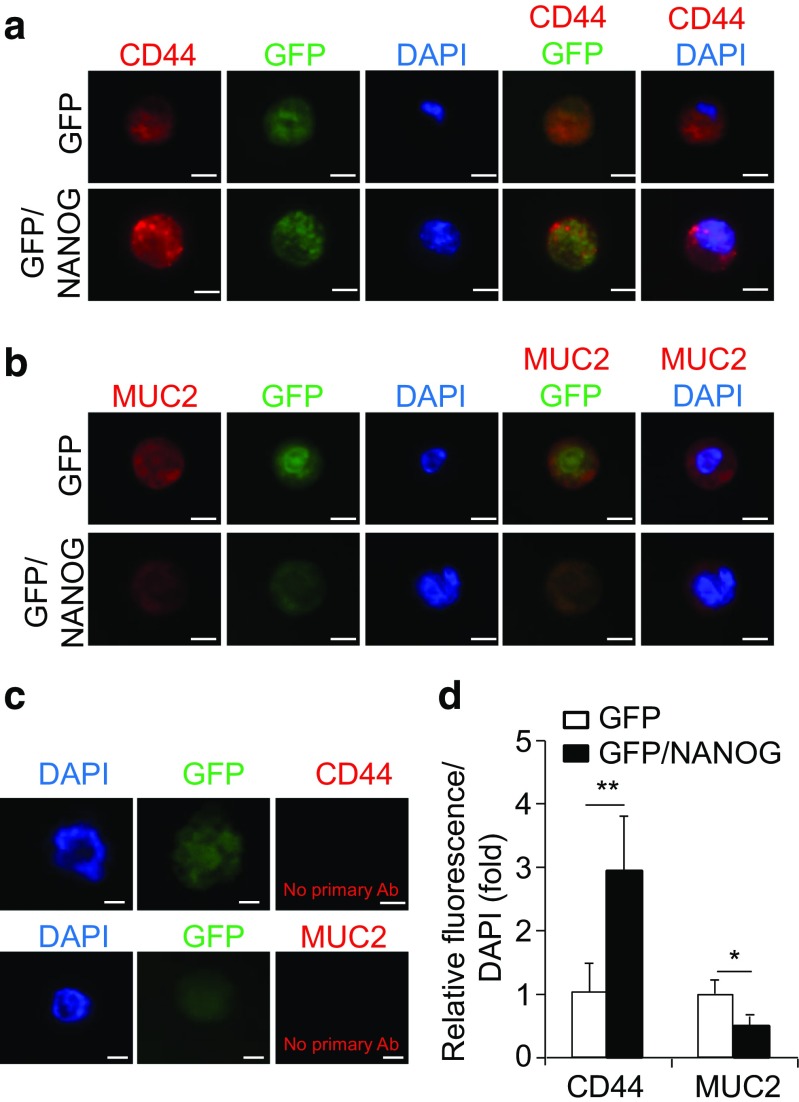
Immunofluorescence staining of CD44 and MUC2 in the HCT116 GFP versus HCT116 GFP/NANOG colonospheres. **a** An apparent increase of CD44 expression level (*red*) in GFP/NANOG-derived colonosphere while (**b**) the expression level of MUC2 (*red*) in GFP/NANOG-derived colonosphere was lower. **c** Negative controls, the primary antibodies were replaced with BSA. **d** Quantitative data showing the expression level of CD44 and MUC2 in GFP versus GFP/NANOG colonospheres. *Scale bars* =10 μm, 40× magnification. Data represent mean ± SD *n* = 3 independent experiments. Student *t* test was used to calculate *p* values (*P < 0.05, **P < 0.01, ***P < 0.001)

Stem-like self-renewal and differentiation capacities of colonospheres can also be examined by immunofluorescence assay. In this study, colonospheres were stained for the CD44 stemness marker (Fig. 4a) and MUC2 differentiation marker (Fig. 4b) along with negative controls for primary antibodies (i.e. without primary antibody) and using the CD44-siRNA knockdown cells (Fig. 4c and data not shown), following the steps illustrated in the above protocol. Colonosphere derived from HCT116-GFP/NANOG cells also showed increased CD44-expression (Fig. 4d) compared with the differentiation marker MUC2 (Fig. 4d).

Among different published protocols there is considerable variability which may influence the formation efficiency and other properties of spheres [20, 37, 40]. As outlined above, we established spheroid formation from human colon cancer cells using DMEM/F12 medium supplemented with N-2, bFGF and EGF. Some of previous reports recommended the use of MEGM supplemented with B-27, bFGF, Heparin and SingleQuots (containing insulin, recombinant epidermal growth factor (rEGF) and hydrocortisone), while some added only B-27 and rEGF. These protocols were assessed using different conditions in different cell lines but no significant difference in spheroid formation was observed in these cells [36, 38, 39].

Below are tips for troubleshooting which may help increase high colonosphere formation efficiency. First, start the experiment with low-passage cell line, and limit the number of passaging. We use CRC cell lines of up to 10–12 passages (up to 2 months of in vitro culture). Another factor is the activity of growth factors; N-2, EGF and bFGF are added to stem cell medium immediately before use, as these growth factors may quickly undergo degradation in the medium. Furthermore, Poly-L-lysine is a charge enhancer, and therefore, it can be used for coating many surfaces as it contains L- isomer for cell attachment. However, as outlined above, we have chosen to coat coverslips with poly-L-Lysine while other protocols reported coating with gelatine instead [41, 42].

One major advantage of using this particular protocol is that colonospheres are generated directly on coverslips from the beginning of the experiment; whereas other protocols generate colonospheres for 2 weeks in plates and then transfer them to the coverslips, which requires more time. However, the current protocol has a number of limitations. Because colonospheres are formed in a very small fraction, to obtain high number of colonospheres for large scale experiments, may require using a lot of expensive stem cell growth medium. Furthermore, primary colonospheres formed over a period of 10 days to 2 weeks of incubation in culture. Maybe using of new recombinant agents and a co-culture system with colonic myofibroblasts that could promote stemness activity, can decrease the time of colonospheres formation. Moreover, freshly prepared medium is added to the colonosphere culture every 3–4 days, therefore there is possibility to lose the colonospheres formed while changing media, since colonospheres are unattached floating spheroid colonies. While several CRC cell lines have been shown to form colonosphere using this protocol, there may be exceptions. However, the current protocol is restricted to the CRC cells; in the future it might be possible to examine other epithelium-derived cancer cell lines.

Furthermore, following this protocol, HCT116 GFP/NANOG cells display a higher expression of CD44 relative to HCT116 GFP cells, whereas it is the opposite for MUC2. In line with this, NANOG is one of the transcriptional factors that been revealed to characterize colorectal CSCs as well as is important for embryonic stem cell pluripotency and differentiation [43, 44]. Therefore, inhibition of NANOG might induce differentiation of CSCs into non-stem cancer cells. This is consistent with recent reports that differentiation and dedifferentiation of cancer cells might be induced by the tumor microenvironment in addition to genetic mutation of normal stem cells.

Taken together, spheroid-forming assays have gained a wide popularity in cancer stem cell research and for a wide range of human tumor cells [45, 46]. Under the experimental condition with a stem cell medium, only cancer cells with self-renewal ability are expected to grow and maintain their spheroid morphology. The protocol defined here presents an efficient method for enriching cultures of CRC cells with stem cell features and can be applied in a wide range of cancer primary and immortalised cell lines. In this manner, a variety of different cell surface markers and signaling pathways can be assessed for their influence on CSCs phenotype. In conclusion, the colonosphere assay presented in this protocol is a valuable tool for investigating the cellular and molecular pathway(s) essential for the growth and maintenance of self-renewal of CSCs [32] and cell fate decision, as well as cell-cell interactions.
